# COVID-19 and ophthalmology: an underappreciated occupational hazard

**DOI:** 10.1017/ice.2020.238

**Published:** 2020-05-15

**Authors:** Irene C. Kuo, Terrence P. O’Brien

**Affiliations:** 1 Wilmer Eye Institute, Johns Hopkins University School of Medicine, Baltimore, Maryland; 2 Bascom Palmer Eye Institute, University of Miami Miller School of Medicine, Miami, Florida

## Abstract

The proximity required of a thorough biomicroscopic slit-lamp examination may put ophthalmologists at increased risk for respiratory-borne infection with SARS-CoV-2. Conjunctivitis has been described in a few patients with COVID-19 and other coronavirus syndromes. Although SARS-CoV-2 has been detected in the conjunctival secretions or tears of patients with COVID-19 and conjunctivitis, transmission of infection through respiratory droplets to ophthalmologists without eye protection or masks may be the bigger concern.

Although generally considered a surgical subspecialty, ophthalmology involves much “chair time,” involving close contact with patients’ aerosolized respiratory droplets or tears and often manipulation of eyelids and conjunctiva during slit-lamp examination. Few (if any) ophthalmologists routinely wear respiratory masks or gloves unless the patient appears to have a respiratory infection or conjunctivitis with tearing or other discharge. Few are aware that the late Dr Li Wenliang, who alerted Chinese government officials of an incipient viral syndrome that would be named COVID-19, was a young ophthalmologist. By his account, he believed he contracted the infection from a glaucoma patient. It is unclear whether the patient had conjunctivitis^1^ or was “asymptomatic.” A month after exposure, Dr Li, age 34, succumbed to respiratory failure. Many systemic viral infections have ocular manifestations; examples include adenovirus,^2^ enterovirus 70, H1N1, SARS coronavirus (SARS-CoV),^3^ and human coronavirus NL63,^4^ the last of which was, in fact, first identified in a 7-month-old child with conjunctivitis and bronchiolitis.^4^ Therefore, it may not be surprising that ophthalmologist Dr Li wisely deduced that an incipient SARS-like viral outbreak was occurring in Wuhan, China, well in advance of others because coronavirus syndromes are known to be associated with conjunctivitis.

Conjunctivitis can be a clinical manifestation of coronavirus 19 (COVID-19). It was described in the 2 eyes of the single patient with conjunctivitis in a series of 30 patients who had “common” and “severe” forms of COVID-19,^5^ and in the 2 eyes of a patient who went from home quarantine to hospital admission during which time this patient developed bilateral follicular conjunctivitis.^6^ Conjunctivitis was the initial symptom of a third individual who was part of a Chinese expert panel investigating the outbreak in Wuhan and developed COVID-19.^7^ The prevalence of “conjunctival congestion” was 0.8% in 1,099 cases of confirmed COVID-19, but 8 of these 12 patients had severe or critical disease requiring ventilation,^8^ which suggests that they may not have had conjunctivitis. In COVID-19 patients with conjunctivitis, SARS-CoV-2 has been recovered from conjunctival secretions, posing a potential route of ocular transmission.^5,6^ Patients who present to ophthalmologists with isolated conjunctivitis may already be infected with and secreting SARS-CoV-2 in tears and elsewhere, but infected persons can also be asymptomatic or presymptomatic. Thus, universal precautions that should be reflexive standard ophthalmic practice apply more than ever. However, the risk of transmission through tears or conjunctival secretion is probably small relative to respiratory transmission.

With the COVID-19 outbreak, caused by severe acute respiratory syndrome coronavirus 2 (SARS-CoV-2), is a concern that ophthalmologists may be inherently at increased occupational risk for infection. The essential nature of encounters in ophthalmology clinics necessitates patient and ophthalmologist to be separated by <20 cm during slit-lamp biomicroscopic examination and <5 cm in situations requiring direct ophthalmoscopy, yet the World Health Organization recommended “1 meter (3 feet) distance between yourself and anyone who is coughing or sneezing” and the Centers for Disease Control and Prevention recommended 6 feet from anyone. “Physical distancing” is not an option for ophthalmologists.

Because of physical proximity, a presymptomatic patient with COVID-19 could transmit disease by breathing or coughing on an ophthalmologist. Another mode of transmission that is underappreciated is that infectious body fluids and aerosol droplets can be transmitted to exposed conjunctiva, which can serve as a portal of entry.^7^ By at least one account, a member of a Chinese national expert panel on pneumonia reported that he was infected via ocular exposure during an inspection in Wuhan. He was wearing an N95 mask but no eye protection. Several days before he developed COVID-19, he developed bilateral redness of his eyes.^7^ In the 2003 Toronto SARS outbreak, 7 of 45 laboratory-confirmed SARS patients requiring intubation transmitted SARS-CoV to 26 of 697 healthcare workers.^9^ The highest estimated healthcare worker risk in generalized estimating equation models was eye or mucous membrane exposure to body fluids (odds ratio, 7.3), while in the classification and regression trees analysis, the primary healthcare worker related risk factor was whether or not eye protection was worn.^9^ This analysis does not mean that conjunctival contact is a primary mode of spread of SARS-CoV but that the conjunctiva is a possible portal of entry for SARS-CoV and SARS-CoV-2 given the ocular tropism of other respiratory viruses like human coronavirus NL63 and adenovirus.

In a 2004 series of 36 patients with suspected SARS-CoV,^3^ the 3 with SARS-CoV RNA in their tears had sampling performed early in their disease course and whether these patients had conjunctivitis was not specified. Although no one studied whether SARS-CoV was present in convalescent patients or whether tears of infected patients were infectious, ophthalmologists should probably don protective equipment when examining certain groups of patients in the pandemic caused by SARS-CoV-2. With the SARS outbreak in 2004, Singaporean ophthalmologists wrote, “Stringent barrier methods using the ‘M3G’ (mask, gown, gloves, and goggles) should be the gold standard when dealing with suspected SARS patients.”^3^ These recommendations make sense in 2020 for ophthalmologists examining high-risk patients. Hand hygiene should occur before and after every patient encounter. Prior to patient examination, ophthalmologists should perform hand hygiene consisting of alcohol-based hand sanitizer or washing hands with soap and water for 30 seconds. Then they should wear gloves, which should be discarded after every patient encounter, followed by hand hygiene. Wearing a mask and eye protection is also very important for ophthalmologists to prevent viral entry via the conjunctiva or respiratory tract.

It is better to avoid or decrease exposure than try to contain exposures. At the front end of protection of patients and ophthalmology personnel is instituting screening measures prior to patient arrival. Hong Kong provides one example. Starting January 25, 2020, the emergency response level was activated in all public hospitals, and the 2 hospital ophthalmology departments serving the Kowloon peninsula with a catchment area of 1.1 million people adopted even stricter standards to curb disease transmission in ophthalmology patients.^10^ They implemented the following measures: (1) they reduced clinic volume and number of elective procedures via short message service (ie, texts) to patients to postpone nonurgent appointments; (2) they required all ophthalmology personnel to wear face masks; (3) at the clinic entrance, they screened all ophthalmology patients and accompanying persons by infrared thermometers; and (4) they administered questionnaires to afebrile patients inquiring about occupation and travel to affected areas or contact with person(s) traveling to such areas or contact with a suspected or confirmed case within 14 days. They also screened for symptoms of upper respiratory tract infection and acute conjunctivitis. If any answer was affirmative, the patient’s appointment was postponed for at least 14 days. Ophthalmologists performing dacryocystorhinostomy were told to don personal protective equipment when performing nasal endoscopy; general anesthesia cases were re-evaluated given the risk of aerosol generation from endotracheal intubation.^10^

Ophthalmologists face specific occupational risks during this pandemic. At present, conjunctivitis appears to be an infrequent though possibly early manifestation of COVID-19. Contact with an infected patient’s conjunctiva or tears probably is not as likely to transmit disease as contact with aerosolized respiratory secretions. Based on anecdotal reports of SARS-CoV-2 transmission to healthcare professionals without eye protection, the ocular tropism of respiratory viruses, and evidence of SARS-CoV transmission to healthcare workers in the SARS outbreak more than a decade ago, masking and eye protection are important for ophthalmologists. As physicians, ophthalmologists have an essential role in guiding infection control measures to protect other patients, staff, doctors, and the community from unnecessary exposures. Like Dr Li, ophthalmologists may be the first to diagnose a more serious condition than isolated conjunctivitis but more important is the increased risk of transmission given the close proximity of ophthalmologist and patient during ophthalmic examination. And like Dr. Li, without the measures described above, ophthalmologists may face a potentially lethal occupational hazard.
